# Genome-Wide Identification and Expression Analysis of Lipoxygenase Genes in Rose (*Rosa chinensis*)

**DOI:** 10.3390/genes14101957

**Published:** 2023-10-18

**Authors:** Wenqi Dong, Bo Jiao, Jiao Wang, Lei Sun, Songshuo Li, Zhiming Wu, Junping Gao, Shuo Zhou

**Affiliations:** 1 Beijing Key Laboratory of Development and Quality Control of Ornamental Crops, Department of Ornamental Horticulture, China Agricultural University, Beijing 100193, China; dddwqqq@126.com; 2 Hebei Academy of Agriculture and Forestry Sciences, Shijiazhuang 050051, China

**Keywords:** lipoxygenase, rose, JA, aphid

## Abstract

Lipoxygenases (LOX) play pivotal roles in plant resistance to stresses. However, no study has been conducted on *LOX* gene identification at the whole genome scale in rose (*Rosa chinensis*). In this study, a total of 17 RcLOX members were identified in the rose genome. The members could be classified into three groups: 9-LOX, Type I 13-LOX, and Type II 13-LOX. Similar gene structures and protein domains can be found in RcLOX members. The *RcLOX* genes were spread among all seven chromosomes, with unbalanced distributions, and several tandem and proximal duplication events were found among *RcLOX* members. Expressions of the *RcLOX* genes were tissue-specific, while every *RcLOX* gene could be detected in at least one tissue. The expression levels of most *RcLOX* genes could be up-regulated by aphid infestation, suggesting potential roles in aphid resistance. Our study offers a systematic analysis of the *RcLOX* genes in rose, providing useful information not only for further gene cloning and functional exploration but also for the study of aphid resistance.

## 1. Introduction

As a non-heme iron-containing dioxygenase, Lipoxygenase (LOX; EC 1.13.11.12) can catalyze the oxygenation of polyunsaturated fatty acids (PUFAs) with a (1Z, 4Z)-pentadiene system to generate fatty acid hydroperoxides [1]. Generally, depending on the oxygenation site at the 9th or 13th carbon of the PUFA chain, plant LOX members can be classified into 9-LOX or 13-LOX, respectively. The LOX protein contains two conserved structural domains, the PLAT/LH2 (Polycystin-1, Lipoxygenase, α-Toxin/Lipoxygenase Homology) domain at the N-terminal end, which plays an essential role in membrane binding, and the histidine (His)-rich LOX structural domain at the C-terminal end, which functions in exerting enzymatic activity [2,3].

*LOX* genes are widely involved in abiotic stress resistance [4,5], growth and development [6,7], fruit ripening [8], senescence processes [9], wounding [10], jasmonate (JA) biosynthesis [11], and biotic attack. Generally, LOX plays a positive role during responses to biotic stress in plants. Rice with enhanced *OsLOX1* expression was more resistant to brown planthopper attack, with a higher level of JA content [12]. *Lox4* and *lox5* mutants possessed decreased JA levels and showed greater susceptibility to *Fusarium verticillioides* in maize [13]. Additionally, *OsHI-LOX* in rice [14], *TomLoxD* in tomato [15], *ZmLOX10* in maize [16], and *LOX2.2* in barley [17] all played positive roles in resistance to biotic stresses, possibly due to JA biosynthesis. However, *LOX3* is a susceptibility factor for the microbial pathogen *Ustilago maydis* in maize, as *lox3* mutant plants showed significantly decreased susceptibility to this important maize pathogen [18]. These results suggest that the functions of specific *LOX* genes in biotic stress are complex and need further study.

As one of the most popular horticultural plants, rose (*R. chinensis*) is vulnerable to various insects, especially aphids [19], which can cause serious damage and also deliver plant viral diseases to plants such as phloem sap-feeding insects [20]. There are three types of resistance to aphids in plants: antixenosis, antibiosis, and tolerance [21]. In barley, overexpression of *LOX2.2* in plants facilitates lower aphid numbers, as antisense plants maintained higher aphid numbers in short-term fecundity tests, possibly due to the up-regulation of JA-regulated genes [17].

*LOX* genes have been identified at the whole genome scale in many plant species. For example, there are 6 *LOX* genes in Arabidopsis and 14 in rice [22], 8 in pepper [23], 14 in tomato [10], 20 in *Artemisia annua* L. [24], 15 in turnip [25], 11 in tea plant (*Camellia sinensis*) [26], and 13 in maize [27]. In rose, one *LOX* gene, the *Rlox1* transcript, was dramatically induced during petal senescence [28]. However, no study has yet explored *LOX* gene identification at the whole genome scale in rose, as the gene functions of most *RcLOX* members remain unknown.

To explore the potential roles of RcLOX members in rose, especially in response to aphids, in this study, *LOX* gene members were identified in the rose genome using BlastP and HMM search methods, and their chromosome localization, gene structures, protein motifs, isoelectric points, molecular weights, subcellular location, and expression patterns in different tissues and responses to aphid infestation were analyzed. This systematic analysis of the complete sets of *RcLOX* genes will provide useful information for further gene cloning and functional exploration, especially in the study of aphid resistance in rose.

## 2. Materials and Methods

### 2.1. Genome-Wide Identification of LOX Gene Members in Rose

The sequences of LOX members in rose (*R. chinensis*) were obtained using the Hidden Markov Model (HMM) combined with BlastP analysis. The seeds of PF00305 were obtained from Pfam (http://pfam.xfam.org, accessed on 16 June 2023), and the putative RcLOX protein sequences were retrieved from HMMER research (http://hmmer.org/, accessed on 17 June 2023). To further confirm whether the putative RcLOX proteins contained the complete LOX domain and PLAT/LH2 (polycystin-1, lipoxygenase, α-toxin domain or lipoxygenase homology) domain, the putative LOX proteins sequences were submitted to the NCBI Conserved Domains Database (CDD: https://www.ncbi.nlm.nih.gov/cdd, accessed on 17 June 2023) for analysis.

### 2.2. Phylogenetic and Amino acid Sequence Analysis

The amino acid sequences of the LOX members of rose, Arabidopsis (*Arabidopsis thaliana*), rice (*Oryza sativa*), and tomato (*Solanum lycopersicum*) were selected. Multiple sequence alignments of LOXs were analyzed using the DNAman program with the default parameters. The phylogenetic analysis was constructed using Molecular Evolutionary Genetics Analysis (MEGA) version 11.0 with the maximum likelihood estimation tree under the WAG model and γ distributed (G) method, which was tested using the bootstrap method with 1000 replicates [29]. The prediction of subcellular localization was performed using the website https://wolfpsort.hgc.jp/ (accessed on 17 July 2023). The protein molecular weights (MW) and isoelectric points (PI) of RcLOX proteins were calculated with ExPASy (http://expasy.org/, accessed on 17 July 2023).

### 2.3. Chromosomal Location and Collinearity Analysis of RcLOX Genes

Genes were first filtered with the longest transcript. The local alignment was further conducted using Blast. By classifying genes using the program “duplicate_gene_classifier” and collinearity analysis in MCScanX [30], tandem duplication and segmental duplication events were investigated. The chromosomal distribution and collinearity relationship of *RcLOX* genes were visualized using Circos [31].

### 2.4. Gene Structure and Conserved Motifs Identified

The structures of deduced *RcLOX* genes were analyzed in GSDS2.0 (Gene Structure Display Server 2.0: http://gsds.gao-lab.org/, accessed on 20 July 2023), and the conserved motifs contained in deduced RcLOX protein sequences were identified using the MEME website (MEME 5.4.1: https://meme-suite.org/meme/doc/meme, accessed on 20 July 2023). The number of motifs was set at 10. The amino acid sequences were uploaded to Batch CD Search for conserved protein domain analysis (https://www.ncbi.nlm.nih.gov/Structure/bwrpsb/bwrpsb.cgi, accessed on 20 July 2023).

### 2.5. Secondary and Tertiary Structure Prediction of RcLOX Proteins

Secondary structure prediction of RcLOX proteins was conducted via SOPMA (Self-Optimized Prediction Method with Alignment) using the corresponding website (https://npsa-prabi.ibcp.fr/cgi-bin/npsa_automat.pl?page=/NPSA/npsa_sopma.html, accessed on 2 August 2023). For tertiary structure prediction, SWISS-MODEL (https://swissmodel.expasy.org, accessed on 3 August 2023) was used to build homology models. The desired template was selected according to both the identity and QMQE score derived from the Blast and HHblits methods [32,33]. QMEAN scores were used to estimate the quality of the models. Models of 17 RcLOX proteins were then built and viewed in PyMOL v2.5.

### 2.6. Prediction of Cis-Acting Elements in the Promoter of Rose LOX Genes

In total, 2000 bp sequences upstream from the translational start sites of the deduced *RcLOX* genes were recognized as the promoter sequences and extracted from the Ensembl Plants database (http://plants.ensembl.org/index.html, accessed on 1 August 2023). The *cis*-acting elements in promoter sequences were predicted using the online PlantCARE website (http://bioinformatics.psb.ugent.be/webtools/plantcare/html/, accessed on 1 August 2023).

### 2.7. Rose Growth Conditions and Aphid Infestation

In this study, 1-year-old rose (*R. chinensis*, var. *Harmonie*) plants were grown in a greenhouse with a photoperiod of 16/8 h and 22/18 °C Day/night. Lighting was supplied by LED lights, and the light intensity was adjusted to 490–500 µmol m^−2^ s^−1^. Each rose plant was grown in a 25 cm diameter pot filled with soil composed of loam soil, peat, and sand (2:1:1). For the expression analysis of *RcLOX* genes in different tissues, samples of the leaf, stem, root, bud, and flower were collected during the flowering period. For the expression response to aphid infestation, fresh young leaves of rose plants during the floral initiation period were challenged with 20 aphids, and the leaf tissues were collected after 72 h from the aphid-treated and control plants. All samples were frozen in liquid nitrogen immediately and stored at −80 °C.

### 2.8. RNA Extraction and Quantitative RT-PCR Analysis

The total RNA from leaves and flowers was extracted separately using a FastPure Plant Total RNA Isolation Kit (Polysaccharides and Polyphenolics-rich) (Vazyme, RC401-01, Nanjing, China) following the manufacturer’s instructions. The RNA concentrations were measured with a NanoDrop 2000 spectrophotometer. High-quality RNA (1 μg) from each sample was reverse-transcribed using the HiScript RT SuperMix for qPCR (Vazyme, R323-01, Nanjing, China) following the protocol from the manufacturer. Next, qRT-PCR was performed with ChamQ Universal SYBR qPCR Master Mix (Vazyme, Q711-02, Nanjing, China). *RcActin* was used as the internal control gene. The relative expression levels of genes were calculated via the 2^−ΔΔCT^ method. Three biological replicates and three technical replicates were performed for each experiment. The primer sequences are listed in Table S1.

## 3. Results

### 3.1. Identification of the LOX Genes in R. chinensis

Based on the amino acid sequences of the LOX gene family, a total of 23 RcLOX genes were obtained from the *R. chinensis* genome via HMMER research and Blast analysis. After removing six members containing truncated domains via CDD analysis (Table S2), 17 RcLOX genes were identified and named from RcLOX1 to RcLOX17 according to their Gene IDs and structures. Detailed information on the 17 RcLOX genes is provided in Table 1. The number of amino acids (aa) in the 17 RcLOX protein varied from 784 to 981, while the predicted isoelectric points of the encoded proteins varied from 5.48 to 8.19, and molecular weight points ranged from 89,452.09 to 110,595.97. The subcellular localization results showed that most of the RcLOX proteins were localized in the cytoplasm and chloroplast. Eight RcLOX genes were localized in the cytoplasm, including RcLOX1, RcLOX4, RcLOX5, RcLOX6, RcLOX9, RcLOX10, RcLOX13, and RcLOX17. Eight RcLOX genes were localized in the chloroplast, including RcLOX2, RcLOX3, RcLOX7, RcLOX11, RcLOX12, RcLOX14, RcLOX15, and RcLOX16; only RcLOX8 was localized in the nucleus (Table 1).

### 3.2. Phylogenetic Analysis of LOX Members

**Figure 1:**
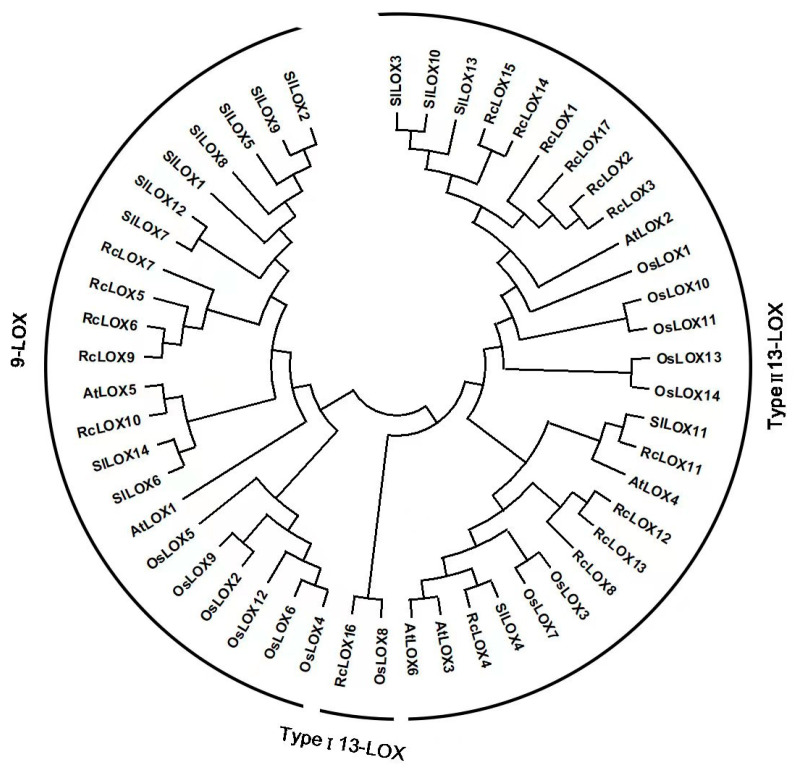
Phylogenetic tree of LOX proteins from rose, Arabidopsis, rice, and tomato. All LOX proteins of rose (17 RcLOX), Arabidopsis (6 AtLOX), rice (14 OsLOX), and tomato (14 SILOX) were divided into three groups, 9-LOX, Type I 13-LOX and Type II 13-LOX.

To explore the phylogenetic relationship of LOX members among the plants, the amino acid sequences of LOX genes from Arabidopsis, rice, tomato, and 17 identified RcLOX genes were selected for phylogeny construction using the MEGA v11.0.11 software with the maximum likelihood method (Table S3). The results showed the evolutionary status and grouping attribution for members of the LOX family. These family members can be sorted into two large groups and one small group, according to the sequence characteristics and clustering analysis. The two largest groups contained 27 and 22 members, belonging to Type II 13-LOX and 9-LOX, respectively. Five RcLOX members (RcLOX5/6/7/9/10) were contained in the 9-LOX group, in which four RcLOXs (RcLOX5/6/7/9) were grouped together, whereas RcLOX10 was separated and homologous to AtLOX5. Eleven RcLOX members (RcLOX1/2/3/4/8/11/12/13/14/15/17) were contained in Type II 13-LOX, in which six RcLOXs (RcLOX1/2/3/14/15/17) were grouped together, while five RcLOXs (RcLOX4/8/11/12/13) were classified into another sub-group. The smallest group, Type I 13-LOX, contained only OsLOX8 and RcLOX16 (Figure 1).

### 3.3. Chromosomal Locations and Collinearity Analysis of RcLOX Genes

**Figure 2:**
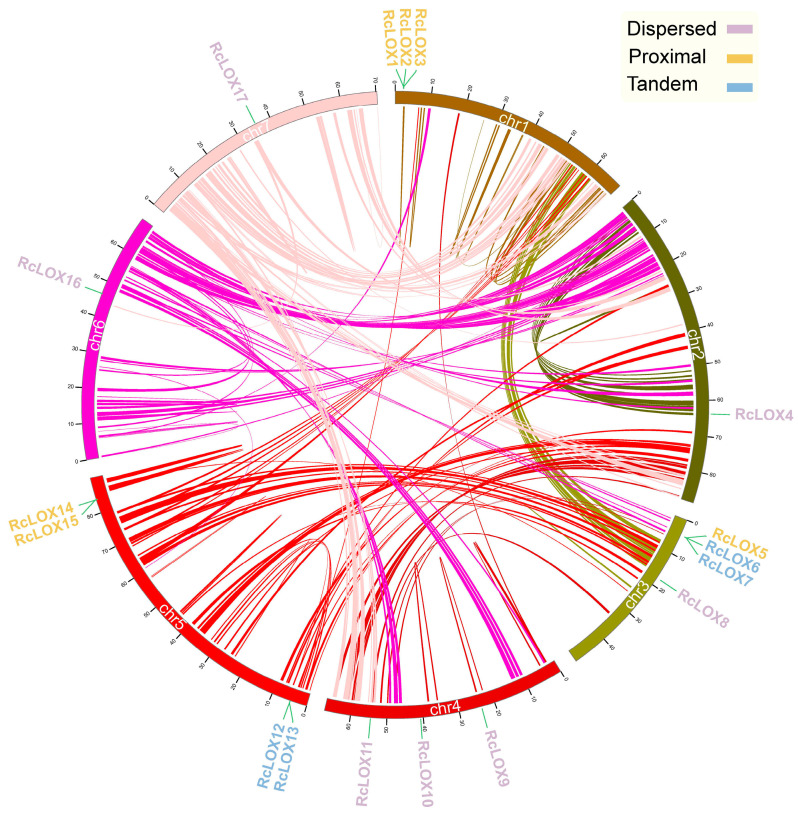
Chromosomal location and collinearity analysis of *RcLOX* genes in the rose genome. The chromosome number is indicated above each chromosome with the scale in megabases (Mb). Different duplication events of *RcLOX* genes are annotated in the upper-right corner. Curves represent the segmental duplication pairs in the whole rose genome.

All seventeen *RcLOX* genes were located on seven chromosomes (2n = 2X = 14) in the *R. Chinensis* genome (Figure 2). Notably, although *RcLOX* genes were not evenly distributed on the chromosomes, they were present on every chromosome. The number of genes located on the chromosomes ranged from one to four, among which Chr3 and Chr5 each contained four *RcLOX* genes; three *RcLOX* genes were observed on chr1 and chr4; and only one *RcLOX* gene was observed on chr2, chr6, and chr7.

We further analyzed the collinearity relationships among the *RcLOX* members. Of all 50,134 genes found in the whole genome, only 3328 (6.46%) genes were considered to be collinear gene pairs. The duplication events of the family proteins were conducted under the program “duplicate_gene_classifier” and visualized via Circos. Several tandem and proximal duplication events were found among RcLOX members. The gene pairs *RcLOX6*, *RcLOX7*, *RcLOX12*, and *RcLOX13* were considered tandem duplications. *RcLOX1*, *RcLOX2*, and *RcLOX3*; *RcLOX5* and *RcLOX6*; and *RcLOX14* and *RcLOX15* were considered proximal duplications due to their high similarity and close distance (Figure 2). The results indicated that RcLOX genes might largely originate from tandem and proximal duplication during revolution.

### 3.4. Amino Acid Sequence, Conserved Motifs Analysis, and Gene Structure Analysis of RcLOX Genes

**Figure 3:**
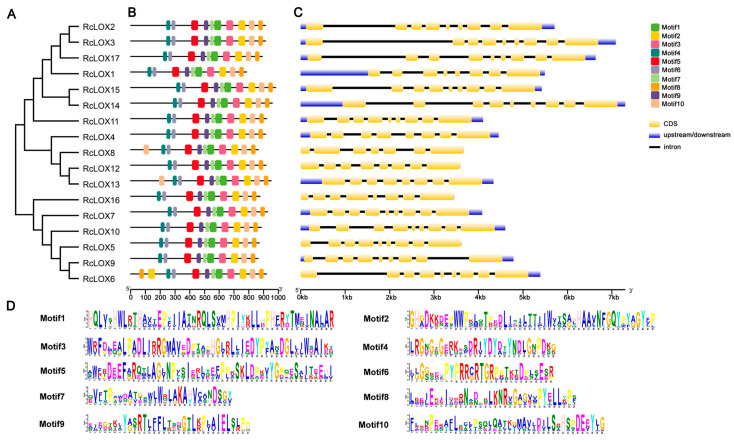
Gene structure and conserved protein motifs of RcLOX proteins in the rose genome. (**A**) The phylogenetic tree of RcLOX proteins formed by using the NJ method. (**B**) Conserved motifs in RcLOX proteins. Boxes of different colors indicate different conserved motifs. (**C**) Gene structure of *RcLOX* genes. The untranslated regions (upstream and downstream), introns, and CDSs are indicated by blue boxes, solid gray lines, and yellow boxes, respectively, with the scale at the bottom. (**D**) Details of 10 conserved motifs.

The amino acid sequences of 17 RcLOXs were analyzed online using MEME, and a total of 10 motifs were selected for analysis. The results revealed that each of the 17 RcLOXs contained 10 conserved motifs. RcLOX8 and RcLOX13 had an extra motif10 in the N terminal of the sequence, and RcLOX6 also had an additional motif8 and motif2 in the N terminal (Figure 3B). Additionally, the exon–intron coding sequence structures were investigated. The results showed that 11 *RcLOX* genes (*RcLOX2/3/5/6/7/9/10/11/14/15/17*) contain eight introns, five *RcLOXs* (*RcLOX1/4/12/13/16*) contained seven introns, and one *RcLOX* (*RcLOX8*) contained six introns (Figure 3C).

### 3.5. The Prediction of Secondary and Tertiary Structural Features of RcLOX Proteins

Secondary structure analysis of the 17 RcLOX proteins was performed using SOPMA and shown in Table S4. All 17 proteins consisted of only four secondary structures, including an α helix, random coil, extended strand, and β turn. Together, the α helix and random coil accounted for more than 80% of the total.

**Figure 4:**
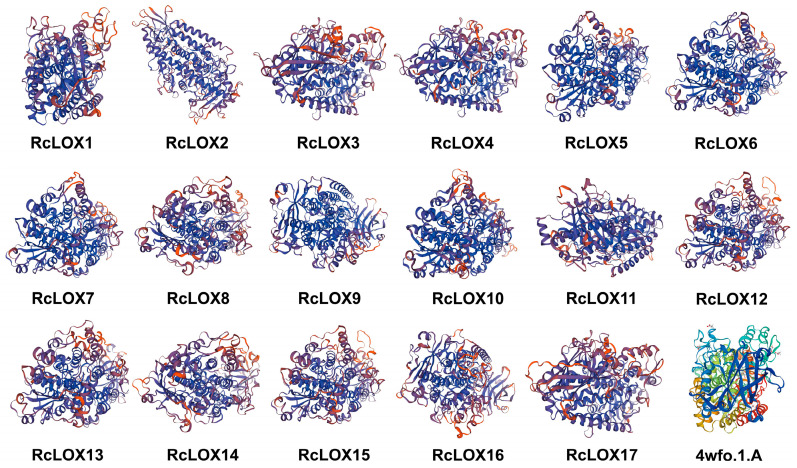
The tertiary structure of the 17 RcLOX proteins. Homology model-building was conducted using the SWISS-MODEL, and 4wfo.1.A was selected as the optimal model template.

For tertiary structural analysis, 4wfo.1.A (manganese-substituted soybean lipoxygenase-1) was selected for the optimal model template based on the identity and QMQE score derived using the Blast and HHblits methods. The three-dimensional structures of RcLOX proteins were further built using an experimentally validated template. The results showed that the tertiary structures of RcLOX proteins were similar (Figure 4). The QMEAN scores, showing the reliability of the estimation, were all over 0.7 for the 17 3D structures predicted in our analysis.

### 3.6. Cis-Acting Elements Prediction in the Promoter of RcLOX Genes

**Figure 5:**
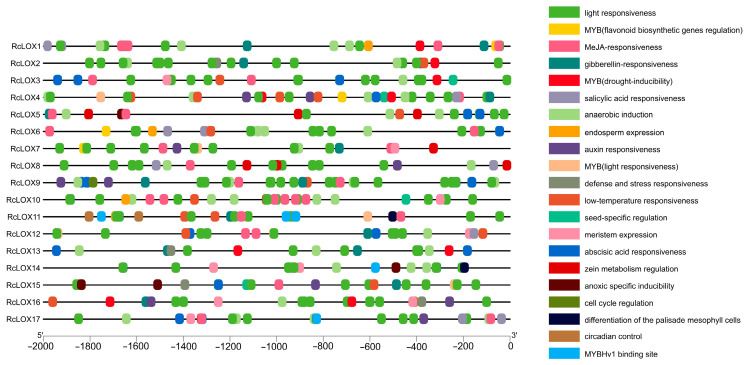
*Cis*-element distributions in the putative promoters of *RcLOX* genes. Boxes of different colors indicate different *cis*-elements.

*Cis*-acting elements are molecular regulate switches on the promoter for the transcriptional regulation of genes. To better understand the regulatory mechanisms of *RcLOX* genes during the process of plant growth, development, and stress response, the 2000 bp regions upstream from the translational starting sites of 17 *RcLOX* genes were analyzed (Figure 5). Using PlantCARE tool analysis, many *cis*-elements involved in hormone and stress responses were identified in the promoters of the *RcLOX* genes. Hormone responsive elements included abscisic acid responsiveness (ABRE), auxin responsiveness (AuxRR-core/TGA-element), gibberellin responsiveness (P-box/GARE-motif/TATC-box), MeJA responsiveness (TGACG-motif/CGTCA-motif), and salicylic acid responsiveness (TCA-Element). Stress-related elements included anaerobic induction (ARE), defense and stress responsiveness (TC-rich repeats), low-temperature responsiveness (LTR), drought-inducibility (MYB binding site, MBS), and light responsiveness. Some *cis*-acting elements related to plant biosynthesis and development, including flavonoid biosynthetic gene regulation (MYB binding site, MBSI), seed-specific regulation (RY-element), zein metabolism regulation (O2-site), cell cycle regulation (MSA-like), endosperm expression/negative expression (GCN4-MOTIF/AACA-motif), and differentiation of the palisade mesophyll cells (HD-Zip1), were also detected on the promoters. The ABRE motif was detected in the promoters of 12 *RcLOXs* (*RcLOX3/4/5/6/9/10/11/12/13/14/15/17*), suggesting that these promoters were involved in potential responses to ABA. Light-responsiveness elements were discovered on every promoter of the *LOX* genes. Several genes (*RcLOX2/4/7/11/12/15*) even contained the specific light-responsive element site for MYB binding. The MYB binding site involved in drought-inducibility was found in the promoters of *RcLOX1/2/3/4/13/16*, indicating that such promoters participated in the drought-stress responses of plants.

### 3.7. The Expression Patterns of RcLOX Genes in Different Tissues

**Figure 6:**
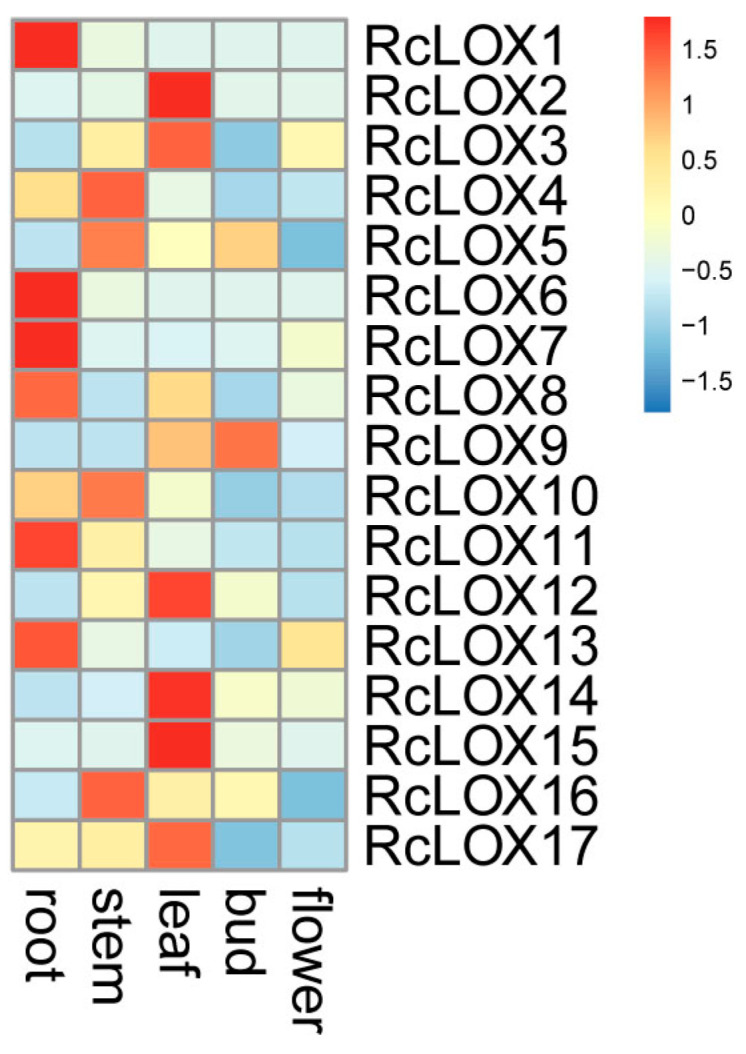
The expression patterns of *RcLOX* genes in five tissues. The heatmap illustrates the expression patterns of *RcLOX* genes in five tissues (root, stem, leaf, bud, and flower). Blue or red indicates lower or higher expression levels of each transcript in each sample, respectively.

The expression of *RcLOX* genes was detected in five tissues (leaf, stem, root, bud, and flower). The expression of *RcLOX* genes showed tissue specificity, indicating their specific functions. *RcLOX2/3/12/14/15* genes were mainly expressed in the leaf, *RcLOX4/5/10/16* in the stem, *RcLOX1/6/7/811/13* in the root, and *RcLOX9* in the bud. However, no *RcLOX* gene was mainly expressed in the flower (Figure 6).

### 3.8. The Expression Patterns of RcLOX Genes in Rose Leaves after Aphid Infestation

**Figure 7:**
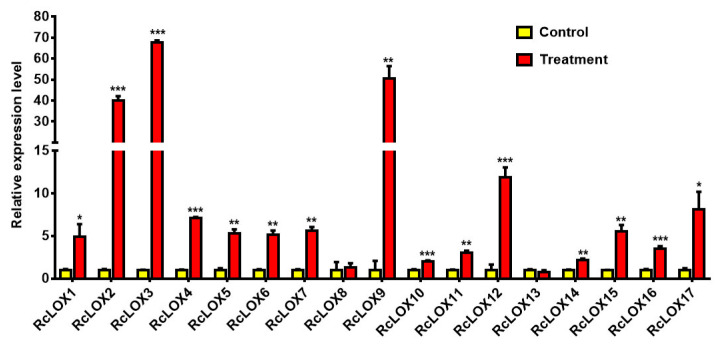
The relative expression levels of *RcLOX* genes responses to aphid infestation in rose leaves. The control and treatment indicate the rose leaves with and without aphid infestation. Statistical significance (*p*-value) is represented by the number of asterisks (“*” for *p* < 0.05, “**” for *p* < 0.01 and “***” for *p* < 0.001).

The responses of 17 *RcLOX* genes to aphid infestation were analyzed. The results showed that 15 out of 17 *RcLOX* genes were up-regulated after aphid infestation in rose, especially *RcLOX3*, *RcLOX9*, *RcLOX2*, and *RcLOX12*, which yielded 67.6-, 50.6-, 40.1-, and 11.9-fold up-regulation responses, respectively, to aphid infestation compared with the control. Eleven other *RcLOX* genes, *RcLOX17*, *RcLOX4*, *RcLOX7*, *RcLOX15*, *RcLOX5*, *RcLOX6*, *RcLOX1*, *RcLOX16*, *RcLOX11*, *RcLOX14*, and *RcLOX10*, were up-regulated with 7.1-, 5.6-, 5.6-, 5.3-, 5.2-, 5.1-, 3.5-, 3.1-, 2.2-, and 2.1-fold responses to aphid infestation, respectively, compared with the control. However, the expressions of *RcLOX8* and *RcLOX13* presented no significant change after aphid infestation (Figure 7).

## 4. Discussion

In this study, a total of 17 *RcLOX* genes were identified in rose (Table 1), which is a greater number of genes than those found in most plant species, e.g., 6 *LOX* genes in Arabidopsis, 14 in rice [22], 8 in pepper [23], 14 in tomato [10], 15 in turnip [25], 11 in tea plant (*C. sinensis*) [26], and 13 in maize [27]. However, the *LOX* gene numbers in rose were lower than the 20 found in *Artemisia annua* L. [24] and 36 found in cultivated peanut (*Arachis hypogaea*) [34]. This result shows that the numbers of *LOX* genes are not proportional to genome size, suggesting that the *LOX* genes underwent changes during evolution. The *RcLOX* genes were divided into three groups, 9-LOX, Type 1 13-LOX, and Type II 13-LOX (Figure 1), consistent with previous results in other species [24,34,35], indicating that the *LOX* gene family is conservative among plant species.

Gene duplication is a fundamental process during genome evolution and is likely to be important for adaptive evolution to changing environments [36]. Additionally, duplicated genes were significantly enriched in resistance-related pathways [37]. This result showed that several tandem and proximal duplication events could be found among *RcLOX* members (Figure 2), consistent with the perspective that *LOX* genes are associated with abiotic and biotic stresses [38]. In this study, the tandem gene pairs *RcLOX6/7,* and proximal gene pairs *RcLOX14/15* showed similar tissue-specific expression patterns, while the expression patterns of *RcLOX12/13*, *RcLOX1/2/3*, and *RcLOX5/6* varied in tissues (Figure 6), suggesting that a functional differentiation of tandem and proximal *RcLOX* genes occurred during evolution. In particular, *RcLOX12* expression was up-regulated significantly after aphid infestation, while the expression of its tandem gene *RcLOX13* presented no change (Figure 7), suggesting different functions in aphid resistance.

Notably, there are many light-responsive *cis*-elements in the promoters of *RcLOX* genes (Figure 5), suggesting an interplay between light and LOX enzymes. The results indicated that the activation of LOX can be induced via excess red light during the plant defense response, which was mediated by phytochrome B [39]. Additionally, light can promote JA biosynthesis to regulate photomorphogenesis in Arabidopsis [40]. Considering that LOX is a key enzyme in the JA synthesis pathway [11,15], it can be inferred that *LOX* genes are key regulators during the light-dependent regulation of the JA pathway in plants [41].

Considerable evidence suggests that LOX is involved in plant resistance to abiotic stress. *AfLOX4* from *Amorpha fruticosa* L. [42], AhLOX29 from peanut [34], and CmLOX13 from oriental melon [43] can all significantly enhance drought tolerance in plants. Moreover, MdLOX3 from apple can positively regulate the salt tolerance in apple Calli and Arabidopsis [5]. In this study, we found many stress-responsive *cis*-elements in the promoters of *RcLOX* genes (Figure 6), suggesting the potential roles of *RcLOX* genes in abiotic stress resistance.

JA is widely involved in biotic stresses in plants. *LOX* genes were found to be induced after aphid infestation in plants [44,45], while *OsHI-LOX* in rice [14], *TomloxD* in tomato [15], and *ZmLOX10* in maize [16] all play positive roles during plant defense against biotic stresses due to JA biosynthesis. In barley, the overexpression of *LOX2.2* maintained lower aphid numbers, and antisense plants had higher aphid numbers [17]. However, the mutation of *lox3* in maize contributed to an enhanced defense response, with a higher PAMP-triggered ROS burst [18], indicating that the LOX enzyme’s involvement in the JA pathway is complicated. In sorghum, aphid infestation can enhance JA biosynthesis, but the exogenous application of JA caused enhanced feeding and aphid proliferation [46]. This result indicates that the involvement of JA in aphid resistance and other biotic stresses is also complicated.

In our study, 15 out of 17 *RcLOX* genes were up-regulated after aphid infestation, including 9-LOX and 13-LOX (Figure 7), suggesting that the functions of RcLOXs in aphid resistance are associated with the 13-LOX-derived JA signal and 9-LOX-derived products [38]. Considering the complexity of JA biosynthesis and signal transduction in resistance to biotic stresses, more research should be conducted to determine the molecular mechanism of LOX underlying aphid resistance in rose.

## 5. Conclusions

In summary, a total of 17 RcLOX members were identified in the rose genome and can be classified into three groups: 9-LOX, Type I 13-LOX, and Type II 13-LOX. Similar gene structures and protein domains can be found in RcLOX members. The *RcLOX* genes were spread among all seven chromosomes with unbalanced distributions. Additionally, several tandem and proximal duplication events were found among *RcLOX* members. Expression of the *RcLOX* genes was tissue-specificity, while every *RcLOX* gene could be detected in at least one tissue. The expression levels of most *RcLOX* genes can be up-regulated via aphid infestation, indicating their potential role in aphid resistance. The present study offers a systematic analysis of the *RcLOX* genes in rose, providing useful information not only for further gene cloning and functional exploration but also for the study of aphid resistance.

## Figures and Tables

**Table 1:** The basic information on *RcLOX* genes in rose.

Gene Name	Gene ID	Ensemble ID	Chr Location	Subcellular Localization	Isoelectric Point	Molecular Weight (Da)	Protein Length (aa)
*RcLOX1*	A0A2P6S733_ROSCH	RchiOBHm_Chr1g0314111	Chr 1: 1,398,352–1,403,844 reverse	cytoplasm	5.68	89,452.09	784
*RcLOX2*	A0A2P6S713_ROSCH	RchiOBHm_Chr1g0314131	Chr 1: 1,418,573–1,424,284 reverse	chloroplast	6.19	102,764.18	913
*RcLOX3*	A0A2P6S725_ROSCH	RchiOBHm_Chr1g0314151	Chr 1: 1,462,401–1,469,489 reverse	chloroplast	6.22	102,838.21	913
*RcLOX4*	A0A2P6RYR4_ROSCH	RchiOBHm_Chr2g0145961	Chr 2: 63,721,112–63,725,566 reverse	cytoplasm	7.33	103,318.16	914
*RcLOX5*	A0A2P6R6Y4_ROSCH	RchiOBHm_Chr3g0455091	Chr 3: 4,945,765–4,949,386 forward	cytoplasm	6.17	99,007.46	870
*RcLOX6*	A0A2P6R6Z5_ROSCH	RchiOBHm_Chr3g0455111	Chr 3: 4,971,165–4,976,556 forward	cytoplasm	5.62	104,221.55	919
*RcLOX7*	A0A2P6R730_ROSCH	RchiOBHm_Chr3g0455121	Chr 3: 4,979,448–4,983,533 forward	chloroplast	6.82	104,342.04	927
*RcLOX8*	A0A2P6RBM1_ROSCH	RchiOBHm_Chr3g0472481	Chr 3: 18,384,687–18,388,356 forward	nucleus	6.27	98,052.53	864
*RcLOX9*	A0A2P6QTX2_ROSCH	RchiOBHm_Chr4g0404751	Chr 4: 24,642,061–24,646,849 forward	cytoplasm	6.41	97,561.23	862
*RcLOX10*	A0A2P6QWV3_ROSCH	RchiOBHm_Chr4g0416591	Chr 4: 41,590,100–41,594,705 reverse	cytoplasm	5.69	100,734.62	884
*RcLOX11*	A0A2P6R0J4_ROSCH	RchiOBHm_Chr4g0430941	Chr 4: 55,302,376–55,306,479 forward	chloroplast	8.19	103,725.45	920
*RcLOX12*	A0A2P6Q438_ROSCH	RchiOBHm_Chr5g0008501	Chr 5: 5,436,496–5,440,087 forward	chloroplast	6.56	104,212.73	916
*RcLOX13*	A0A2P6Q441_ROSCH	RchiOBHm_Chr5g0008511	Chr 5: 5,459,688–5,464,027 forward	cytoplasm	6.78	107,499.38	950
*RcLOX14*	A0A2P6QM50_ROSCH	RchiOBHm_Chr5g0078061	Chr 5: 83,928,147–83,935,447 reverse	chloroplast	5.77	108,405.14	958
*RcLOX15*	A0A2P6QM53_ROSCH	RchiOBHm_Chr5g0078091	Chr 5: 83,993,484–83,998,908 reverse	chloroplast	5.48	110,595.97	981
*RcLOX16*	A0A2P6PTU3_ROSCH	RchiOBHm_Chr6g0282631	Chr 6: 45,898,666–45,902,121 forward	chloroplast	7.9	99,420.98	875
*RcLOX17*	A0A2P6PBW3_ROSCH	RchiOBHm_Chr7g0216951	Chr 7: 34,853,149–34,859,784 reverse	cytoplasm	5.64	94,662.09	891

## Data Availability

Data are contained within the article or Supplementary Material.

## Supplementary Materials

The following supporting information can be downloaded at https://www.mdpi.com/article/10.3390/genes14101957/s1. Table S1: RT-PCR primers for 17 *RcLOX* genes; Table S2: Six removed truncated RcLOX genes; Table S3: LOX gene members from various plant species; Table S4: Secondary structures of 17 RcLOX proteins.
